# Walking on Mild Slopes and Altering Arm Swing Each Induce Specific Strategies in Healthy Young Adults

**DOI:** 10.3389/fspor.2021.805147

**Published:** 2022-01-25

**Authors:** Mary-Elise MacDonald, Tarique Siragy, Allen Hill, Julie Nantel

**Affiliations:** Faculty of Health Sciences, School of Human Kinetics, University of Ottawa, Ottawa, ON, Canada

**Keywords:** gait, stability, posture, arm swing, uphill, downhill

## Abstract

Slopes are present in everyday environments and require specific postural strategies for successful navigation; different arm strategies may be used to manage external perturbations while walking. It has yet to be determined what impact arm swing has on postural strategies and gait stability during sloped walking. We investigated the potentially interacting effects of surface slope and arm motion on gait stability and postural strategies in healthy young adults. We tested 15 healthy adults, using the CAREN-Extended system to simulate a rolling-hills environment which imparted both incline (uphill) and decline (downhill) slopes (± 3°). This protocol was completed under three imposed arm swing conditions: held, normal, active. Spatiotemporal gait parameters, mediolateral margin of stability, and postural kinematics in anteroposterior (AP), mediolateral (ML), and vertical (VT) directions were assessed. Main effects of conditions and interactions were evaluated by 2-way repeated measures analysis of variance. Our results showed no interactions between arm swing and slope; however, we found main effects of arm swing and main effects of slope. As expected, uphill and downhill sections of the rolling-hills yielded opposite stepping and postural strategies compared to level walking, and active and held arm swings led to opposite postural strategies compared to normal arm swing. Arm swing effects were consistent across slope conditions. Walking with arms held decreased gait speed, indicating a level of caution, but maintained stability comparable to that of walking with normal arm swing. Active arm swing increased both step width variability and ML-MoS during downhill sections. Alternately, ML-MoS was larger with increased step width and double support time during uphill sections compared to level, which demonstrates that distinct base of support strategies are used to manage arm swing compared to slope. The variability of the rolling-hills also required proactive base of support changes despite the mild slopes to maintain balance.

## Introduction

Everyday walking environments are complex as they vary in levelness and regularity (Allet et al., 2008). Challenging terrains require gait pattern modifications, through changes in spatiotemporal gait characteristics, kinematics, and kinetics, to accommodate the mechanical constraints. Responses to challenging terrain by the postural control system can be seen in adjustment of spatiotemporal gait characteristics. Compensatory changes such as increased double-support time or step width are a means of coping with uphill or downhill slopes, respectively (Kawamura and Tokuhiro, 1991; Sun et al., 1996; Gottschall and Nichols, 2011). The effectiveness of such changes may be determined by additionally quantifying stability. For example, taking wider steps has been linked to increased mediolateral margin of stability (ML-MoS) (McAndrew Young and Dingwell, 2012), indicating enhanced stability. Vieira et al. (2017) found downhill walking decreased ML-MoS and uphill walking increased ML-MoS compared to level walking, but not all concomitant gait strategies were explored.

The Americans with Disabilities Act mandates that sidewalks have a slope of <2.86°, and ramps be <4.76° (United States, 2010). Thus, everyday uneven terrain includes slight slopes ranging from 0 to 3°, yet most sloped walking studies examined larger and continuous slopes (3–20°) rather than smaller varying slopes (Sun et al., 1996; Leroux et al., 2002; Minetti et al., 2002; Prentice et al., 2004; Lay et al., 2006; Silverman et al., 2012; Kimel-Naor et al., 2017). Investigations of continuous 3° slope (Finley and Cody, 1970; Kawamura and Tokuhiro, 1991; Sun et al., 1996) found no differences between gait walking uphill compared to downhill. However, Prentice et al. (2004) found that stepping onto a 3° incline from level required modified swing limb kinematics, such as increased lower extremity joint flexion, and increased trunk forward inclination (Prentice et al., 2004). Recently, a rolling-hills (-3 to +3°) condition was used to simulate destabilizing terrain (Sinitski et al., 2015, 2019), but uphill and downhill steps were not examined separately despite the unique postural strategies required for each (Leroux et al., 2002).

During walking, the natural 1:1 contralateral arm-leg swing pattern reduces gait's metabolic cost by controlling angular momentum about the vertical axis of the center of mass (COM) (Meyns et al., 2013). This antiphase arm-leg swing pattern can be modulated by adjusting either arm motion or leg motion, which demonstrates the bidirectional nature of this relationship (Bondi et al., 2017). Different arm swing strategies have been shown to have unique impacts on gait stability. For example, walking with arms held may improve stability by increasing trunk inertia which limits CoM movement (Bruijn et al., 2010; Pijnappels et al., 2010). Conversely, some studies found decreased postural control and increased metabolic cost when walking without arm swing (Collins et al., 2009; Punt et al., 2015; Yang et al., 2015), or no difference in postural control between absent and normal arm swing (Bruijn et al., 2010; Hill and Nantel, 2019; Siragy et al., 2020). Alternatively, active arm swing may increase stability by more aptly counterbalancing torques that act on the COM's trajectory (Nakakubo et al., 2014; Punt et al., 2015; Yang et al., 2015; Wu et al., 2016). However, active arm swing's contribution to walking stability remains conflicting (Collins et al., 2009; Bruijn et al., 2010; Meyns et al., 2013; Siragy et al., 2020), especially when walking on challenging terrains.

The purpose of this study was to examine the effect of arm swing on spatiotemporal gait parameters, margin of stability, and postural strategies during uphill and downhill sections of a rolling-hills terrain. We expected that walking on slopes (uphill or downhill sections) with arms held would have compound increases in compensatory gait strategies that may increase stability, while the gait changes from active arm swing would conflict with the compensatory strategies used to navigate sloped walking.

## Methodology

Fifteen healthy adults (8 male, 7 female; age 23.4 ± 2.8 years; height 170.2 ± 8.1 cm; weight 72.3 ± 13.5 kg) volunteered from the Ottawa area. An a priori power analysis revealed that 12 participants were adequate to achieve power at ß = 0.8. Participants had no neurological or orthopedic disorders affecting gait and no musculoskeletal injuries in the previous 6 months. The study was approved by the Institutional Review Board (University of Ottawa) and the Ottawa Hospital Research Ethics Board; all participants provided written informed consent.

### Data Collection

**Figure 1:**
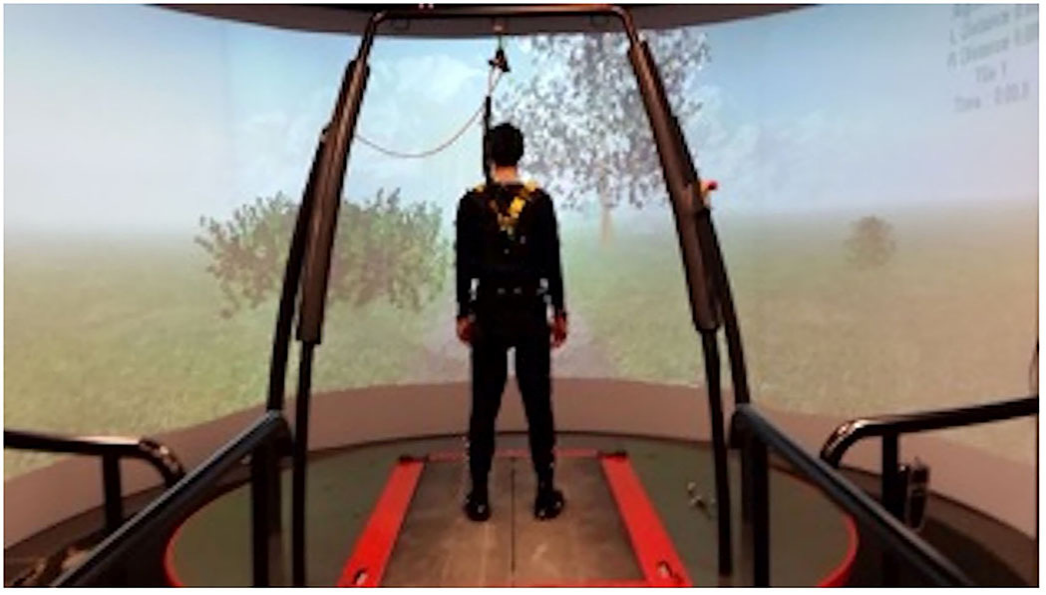
The CAREN-Extended virtual reality system used in this study.

Three-dimensional motion capture was completed using the Computer-Assisted Rehabilitation Environment (CAREN; CAREN-Extended, Motek Medical, Amsterdam, The Netherlands, Figure 1). This system combines a 6 degree-of-freedom platform with integrated split-belt instrumented treadmill (Bertek Corp., Columbus OH), 12-camera VICON motion capture system (Vicon 2.6, Oxford, UK), and 180° projection screen. Participants wore a torso harness attached to an overhead structure when on the treadmill. Platform motion was tracked by three markers, and full body kinematics collected using a 57-marker set (Wilken et al., 2012). Motion data were gathered at a rate of 100 Hz.

### Experimental Protocol

For each trial, participants walked in a virtual park scenario which included a 20 m simulated rolling-hills terrain preceded and succeeded by 40 m of level walking. The rolling-hills terrain was produced by platform oscillations in the sagittal plane (pitch) based on a sum of four sines with frequencies of 0.16, 0.21, 0.24, and 0.49 Hz (Sinitski et al., 2015). Treadmill speed used the self-paced algorithm described by Sloot et al. (Sloot et al., 2014) (Methods 2c) which incorporated anterior–posterior pelvis position, velocity, and acceleration, referenced to the person's initial standing position (heels at the anterior-posterior midline of the treadmill). Visuals on the projection screen matched treadmill and platform conditions in speed and slope.

Trial order was randomized. Separate trials occurred for the three arm conditions: held, normal, and active. Instructions for the held condition were to volitionally hold arms in a still, relaxed position at the participant's sides. For the active condition, participants were instructed that the arms should be roughly horizontal at peak anterior arm swing.

**Figure 2:**
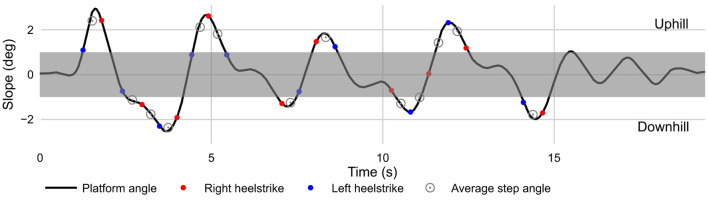
Platform angles throughout the rolling-hills terrain and representative sample of heel-strike gait events and average step angles included in sloped conditions. Average step angles within the shaded region were not counted toward uphill or downhill steps. NB: Figure depicts the AP platform angle throughout the terrain condition, not the elevation of the virtual path.

Uphill sections included steps occurring when the average slope of the platform was between +1 and +3 degrees; downhill sections included steps occurring when the average slope of the platform was between −1 and −3 degrees (Figure 2). No uphill or downhill steps spanned a peak or trough in the rolling-hills terrain. Level walking included steps from the middle 20 m of the 40 m flat section preceding the rolling-hills terrain.

### Data Analysis

Data were imported into Visual3D (C-Motion, Germantown, MD). Kinematic data were filtered at 10 Hz using a 4^th^ order, zero-lag low-pass Butterworth filter, chosen using a residual analysis approach (Winter, 2009). Heel strike and toe-off gait events were calculated using a velocity-based algorithm as previously described (Zeni et al., 2008) and verified using ground reaction forces. Spatiotemporal parameters included speed, step length, step width, step time, percent double-support time (DST), and coefficients of variation (CoV) for step length, step width, step time, and percent double-support time. Speed was retrieved from D-Flow [Motek Medical, Amsterdam, The Netherlands; (Geijtenbeek et al., 2011)] which served as the control software for the CAREN system; we then averaged the speed over each step. Gait stability was quantified using mediolateral margin of stability (ML-MoS) and ML-MoS CoV using previously reported methods (Hof et al., 2005; Hak et al., 2013; Siragy and Nantel, 2020).

Step length was calculated for each step as the hypotenuse of the vertical and anteroposterior distance between the feet at heel strike of the leading leg. The MoS was calculated bilaterally at both heel strikes and defined as the distance of the Extrapolated Center of Mass (xCoM) to the right/left lateral heel marker:


(1) MoS = Lateral heel marker−xCoM

The formula for xCoM was:


(2) $$xCoM\;=\;CoM_{p}\;+\;\left(\frac{CoM_{v}}{\omega \Theta }\right)$$

Where CoMp = CoM's position, CoMv = CoM's velocity. ωΘ was calculated as:


(3) $$\omega \Theta \;=\;\sqrt{\frac{g}{l}}$$

In this term, *g* = 9.81 m/s^2^ and *l* is the length of the inverted pendulum determtextned as the average distance of the right/left lateral heel marker to the CoM at heel-strikes. Visual 3D was used to calculate the CoM's position and velocity.

Kinematic measures included trunk angle (mid-point of the posterior superior iliac spine markers to C7 compared to global vertical, measured in the AP direction with a larger trunk angle indicating increased forward inclination) and trunk acceleration root-mean-square (RMS) in the ML, AP, and VT directions as a measure of upper body variability [with larger RMS values indicating greater variability (Menz et al., 2003; Marigold and Patla, 2008)]. All data reduction prior to statistical analyses were performed using the Julia programming language (Bezanson et al., 2017) and custom code (MacDonald et al., 2021).

### Statistical Analyses

Separate 2-way repeated measures ANOVAs were used to examine significance between each slope (uphill, downhill) compared to level and across arm (held, normal, active) conditions, as well as potential interactions, for all variables using IBM SPSS Statistics 26 (IBM Analytics, Armonk, USA). Assumption of normality was confirmed using a Shapiro-Wilk test and Greenhouse-Geisser *p* was reported when Mauchly's Test of Sphericity was violated. Significance level was set at *p* < 0.05. A Bonferroni correction was used for *post-hoc* tests.

## Results

No significant interaction effects between arm swing and surface slope were found. Statistical information regarding main effects is included in Tables 1, 2, with significant *post-hoc* findings presented in the following text. See Tables 3, 4 for spatiotemporal results and Table 5 for postural kinematics. Tables including the number and average angle of steps analyzed are included in Supplementary Materials 1, 2, with correlations analyses regarding margin of stability in Supplementary Materials 3, 4.

**Table 1:** Main effects for uphill vs. level walking.

**Uphill vs. Level**		**Arms**			**Slope**			**Arms** **× slope**		
**Variable**		***F*(2, 28)**	***p***	** $\eta_{\text{p}}^{2}$ **	***F*(1, 14)**	***p***	** $\eta_{\text{p}}^{2}$ **	***F*(2, 28)**	***p***	** $\eta_{\text{p}}^{2}$ **
Speed		7.59	**0.007**	0.352	6.73	**0.021**	0.325	0.76	0.478	0.051
Step length	mean	49.32	**<0.001**	0.779	40.50	**<0.001**	0.743	0.85	0.439	0.057
	CoV	6.27	**0.006**	0.309	28.95	**<0.001**	0.674	5.23	**0.012**	0.272
Step width	mean	1.17	0.326	0.077	47.80	**<0.001**	0.773	0.02	0.983	0.001
	CoV	5.53	**0.009**	0.283	3.97	0.066	0.221	0.05	0.956	0.003
Step time	mean	15.02	**<0.001**	0.518	6.32	**0.025**	0.311	3.85	**0.033**	0.216
	CoV	1.08	0.355	0.071	23.90	**<0.001**	0.631	1.54	0.232	0.099
DST	mean	14.34	**<0.001**	0.506	30.91	**<0.001**	0.688	0.00	0.998	0.000
	CoV	0.76	0.475	0.052	3.90	0.068	0.218	0.73	0.490	0.050
ML MOS	mean	14.34	**<0.001**	0.506	27.64	**<0.001**	0.664	0.46	0.636	0.032
	CoV	1.22	0.310	0.080	6.45	**0.024**	0.315	0.01	0.986	0.001
Trunk angle		20.63	**<0.001**	0.596	28.02	**<0.001**	0.667	0.60	0.559	0.041
RMS	AP	56.33	**<0.001**	0.801	9.20	**0.009**	0.396	1.35	0.276	0.088
	ML	1.67	0.206	0.107	0.08	0.780	0.006	0.31	0.734	0.022
	VT	4.30	**0.040**	0.235	0.03	0.858	0.002	0.28	0.759	0.020

*Boldfaced numbers indicate significant main effect*.

**Table 2:** Main effects for uphill vs. level walking.

**Downhill vs. Level**		**Arms**			**Slope**			**Arms** **× slope**		
**Variable**		***F*(2, 28)**	***p***	** $\eta_{\text{p}}^{2}$ **	***F*(1, 14)**	***p***	** $\eta_{\text{p}}^{2}$ **	***F*(2, 28)**	***p***	** $\eta_{\text{p}}^{2}$ **
Speed		7.42	**0.008**	0.346	11.1	**0.005**	0.442	0.49	0.619	0.034
Step length	mean	51.96	**<0.001**	0.788	5.0	**0.042**	0.264	2.03	0.150	0.127
	CoV	2.65	0.088	0.159	15.2	**0.002**	0.521	1.80	0.183	0.114
Step width	mean	1.42	0.259	0.092	0.0	0.992	0.000	0.03	0.970	0.002
	CoV	5.65	**0.009**	0.287	2.5	0.140	0.149	0.26	0.777	0.018
Step time	mean	15.47	**<0.001**	0.525	5.4	**0.036**	0.279	2.05	0.148	0.128
	CoV	0.13	0.881	0.009	27.1	**<0.001**	0.660	0.52	0.598	0.036
DST	mean	13.76	**<0.001**	0.496	1.6	0.227	0.102	0.03	0.970	0.002
	CoV	0.94	0.404	0.063	13.3	**0.003**	0.488	0.79	0.462	0.054
ML MOS	mean	5.63	**0.009**	0.287	3.1	0.098	0.183	0.06	0.941	0.004
	CoV	0.52	0.602	0.036	4.4	0.055	0.239	0.57	0.575	0.039
Trunk angle		18.76	**<0.001**	0.573	7.5	**0.016**	0.349	0.28	0.755	0.020
RMS	AP	49.93	**<0.001**	0.781	4.2	0.060	0.230	2.12	0.139	0.131
	ML	0.04	0.892	0.003	1.4	0.252	0.092	0.69	0.510	0.047
	VT	3.78	0.055	0.213	0.1	0.780	0.006	0.03	0.975	0.002

*Boldfaced numbers indicate significant main effect*.

**Table 3:** Comparison of speeds, spatiotemporal gait parameters, and coefficients of variation (CoV) in the three arm swing conditions (held, normal, active) during uphill, level, and downhill walking.

**Slope**	**Arms**	**Speed (m/s)**	**Spatiotemporal**				**CoV (%)**			
			**Step length (cm)**	**Step width (cm)**	**Step time (s)**	**DST (% stride)**	**Step length**	**Step width**	**Step time**	**DST**
Downhill	Held	**1.13 (0.19)**	**52.6 (7.47)^a^**	20.7 (4.34)	**0.50 (0.05)^a^**	**30.9 (3.84)^a^**	7.83 (3.93)	8.67 (4.12)	5.45 (1.48)	11.3 (2.39)
	Normal	**1.26 (0.21)^b^**	**59.0 (8.48)^a^^,^ ^b^**	19.9 (3.44)	**0.49 (0.03)^a^**	**28.8 (4.37)^a^**	5.92 (2.53)	**10.3 (5.37)^a^**	5.56 (1.82)	11.2 (3.41)
	Active	**1.29 (0.24)^b^**	**67.0 (9.41)^b^**	20.7 (4.56)	**0.55 (0.04)**	**27.5 (4.28)**	5.13 (2.23)	**13.8 (7.51)**	5.18 (1.30)	10.6 (3.44)
Downhill vs. Level		0.005^*^	0.042^*^	0.992	0.036^*^	0.227	0.002^*^	0.140	<0.001^*^	0.003^*^
Level	Held	1.23 (0.19)	56.1 (5.24)	20.8 (4.26)	0.51 (0.04)	31.1 (4.06)	5.26 (2.46)	11.0 (7.40)	3.94 (2.95)	8.84 (3.69)
	Normal	1.33 (0.17)	61.6 (6.27)	19.8 (4.22)	0.51 (0.04)	30.1 (3.40)	4.01 (2.21)	10.9 (4.42)	3.24 (1.61)	7.00 (2.47)
	Active	1.38 (0.18)	68.2 (5.50)	20.7 (4.06)	0.55 (0.04)	27.7 (3.15)	4.38 (3.41)	16.0 (7.25)	3.81 (2.71)	8.49 (3.54)
Uphill vs. Level		0.021^*^	**<** 0.001^*^	**<** 0.001^*^	0.025^*^	**<** 0.001^*^	**<** 0.001^*^	0.066	**<** 0.001^*^	0.068
Uphill	Held	**1.13 (0.19)**	**48.8 (6.74)^a^**	23.2 (4.86)	**0.51 (0.06)^a^**	**33.6 (4.01)^a^**	**12.2 (6.77)**	8.21 (3.52)	7.07 (3.41)	9.49 (3.27)
	Normal	**1.28 (0.22)^b^**	**55.4 (6.60)** ^**a, b**^	22.3 (4.33)	**0.52 (0.04)^a^**	**32.7 (3.95)^a^**	**7.91 (2.55)^b^**	**8.90 (4.42)^a^**	6.41 (2.73)	9.54 (1.99)
	Active	**1.30 (0.26)^b^**	**62.0 (8.52)^b^**	23.0 (5.80)	**0.58 (0.06)**	**30.2 (4.03)**	6.87 (2.56)	**13.7 (9.91)**	5.11 (2.16)	9.34 (4.29)

*Data within each slope are represented as the mean values averaged for all 15 participants (8 male, 7 female), mean (standard deviation). Pairwise comparison p-values of slope conditions (Uphill vs. Level and Downhill vs. Level) from two-way repeated measures ANOVA are presented between surface conditions. Statistical significance set at p <0.05 with Bonferroni correction*.

*Boldfaced numbers highlight significant differences with the following specifications*.

a *Different from Active*.

b *Different from Held*.

* *Different from Level*.

**Table 4:** Comparison of mediolateral margin of stability and coefficient of variability in the three arm swing conditions during uphill, level, and downhill walking.

**Slope**	**Arms**	**ML-MoS (cm)**	**CoV ML-MoS (%)**
Downhill	Held	10.9 (3.22)	31.2 (14.7)
	Normal	**10.6 (2.41)^a^**	36.1 (14.6)
	Active	**11.8 (2.77)**	33.4 (14.4)
Downhill vs. Level		0.098	0.055
Level	Held	10.1 (3.60)	36.6 (16.5)
	Normal	9.86 (4.15)	38.7 (23.3)
	Active	11.4 (3.60)	42.8 (19.4)
Uphill vs. Level		**<** 0.001^*^	0.024^*^
Uphill	Held	12.6 (2.70)	28.1 (7.82)
	Normal	12.6 (2.22)	29.7 (14.4)
	Active	13.0 (3.02)	35.2 (29.0)

*Data within each slope are represented as the mean values averaged for all 15 participants (8 male, 7 female), mean (standard deviation). Pairwise comparison p-values of slope conditions (Uphill vs. Level and Downhill vs. Level) from two-way repeated measures ANOVA are presented between surface conditions. Statistical significance set at p <0.05 with Bonferroni correction*.

*Boldfaced numbers highlight significant differences with the following specifications*.

a *Different from Active*.

* *Different from Level*.

**Table 5:** Comparison of kinematic postural variables in the three arm swing conditions during uphill, level, and downhill walking in the anteroposterior (AP), vertical (VT), and mediolateral (ML) directions; Data within each slope are represented as the mean values averaged for all 15 participants (8 male, 7 female), mean (standard deviation).

**Slope**	**Arms**	**AP**		**VT**	**ML**
		**Trunk angle (**°**)**	**RMS**	**RMS**	**RMS**
Downhill	Held	**7.38 (4.01)^a^**	**1.18 (0.30)^a^**	2.28 (0.59)	1.21 (0.46)
	Normal	**6.65 (4.06)^a^**	**1.54 (0.40)^a^^,^ ^b^**	2.55 (0.61)	1.17 (0.44)
	Active	**4.31 (4.20)**	**2.03 (0.41)^b^**	2.66 (0.84)	1.18 (0.30)
Downhill vs. Level		0.016^*^	0.060	0.780	0.252
Level	Held	8.00 (3.78)	1.16 (0.26)	2.29 (0.50)	1.08 (0.32)
	Normal	7.49 (3.90)	1.46 (0.34)	2.59 (0.62)	1.08 (0.44)
	Active	5.43 (3.53)	1.87 (0.35)	2.67 (0.69)	1.17 (0.31)
Uphill vs. Level		**<** 0.001^*^	0.009^*^	0.858	0.780
Uphill	Held	**9.54 (3.92)^a^**	**1.02 (0.16)^a^**	2.25 (0.68)	1.10 (0.24)
	Normal	**9.14 (3.93)^a^**	**1.31 (0.27)^a^^,^ ^b^**	2.62 (0.66)	1.13 (0.36)
	Active	**6.36 (4.30)**	**1.83 (0.36)^b^**	2.73 (0.99)	1.25 (0.51)

*Pairwise comparison p-values of slope conditions (Uphill vs. Level and Downhill vs. Level) from two-way repeated measures ANOVA are presented between surface conditions. Statistical significance set at p <0.05 with Bonferroni correction*.

*Boldfaced numbers highlight significant differences with the following specifications*.

a *Different from Active*.

b *Different from Held*.

* *Different from Level*.

### Arm Swing During Uphill and Downhill Sections of the Rolling-Hills

In this section, corrected *p*-values for each result are presented in parentheses.

Walking with arms held decreased walking speed compared to normal (*p* ≤ 0.044) and active arm swing (*p* ≤ 0.031). Step length increased with increasing arm swing (*p* ≤ 0.01) and, during uphill sections only, step length CoV was greater when walking with arms held compared to with normal arm swing (*p* = 0.027). Active arm swing increased step width CoV compared to normal (*p* ≤ 0.047). Active arm swing increased step time compared to held (*p* = 0.005) and normal (*p* = 0.001). Active arm swing also decreased double support time compared to held (*p* ≤ 0.001) and normal (*p* ≤ 0.014). During downhill sections only, active arm swing increased ML-MoS compared to normal (*p* = 0.014).

Active arm swing decreased trunk angle compared to held (*p* < 0.001) and normal (*p* ≤ 0.006). AP-RMS magnitude was larger with active arm swing compared to held and normal (*p* < 0.001) and smaller with arms held compared to normal (*p* ≤ 0.003). During uphill sections only, main effects were found for VT-RMS but no *post-hoc* significance.

### Uphill vs. Level

Walking on uphill sections decreased walking speed and step length and increased step width, step time, and double support time compared to level. Uphill walking also increased ML-MoS compared to level. Uphill walking increased step time CoV and decreased step length and ML-MoS CoV. Uphill walking increased trunk angle, and decreased AP-RMS magnitude compared to level.

### Downhill vs. Level

Walking on downhill sections decreased walking speed, step length, and step time compared to level. Downhill walking decreased step length CoV and increased step time CoV and double support time CoV. Downhill walking decreased trunk angle compared to level.

## Discussion

This study investigated the effect of various arm swings on spatiotemporal parameters and postural strategies during uphill and downhill sections of a rolling-hills terrain compared to level walking. Regardless of slope, active arm swing increased step time and decreased double-support and trunk angle, while walking with arms held decreased walking speed and trunk angle. During both uphill and downhill sections, walking speed was consistently slower and caused postural and spatiotemporal changes from level walking despite the slopes being mild. Within downhill sections, active arm swing corresponded to increased ML-MoS compared to normal. Compared to level walking, uphill sections increased step width and ML-MoS.

### Variability of Rolling-Hills Condition Required Proactive Base of Support Changes

When walking on the rolling-hills terrain, the magnitude and timing of surface fluctuations was unpredictable (oscillating between −3° and +3°) and required participants to navigate continuous changes in surface slope. For example, a posterior tilt in the surface shifting to an incline may interfere with a leg in late swing and precipitate unplanned foot contact, and an anterior tilt to a decline may induce a stepping response to catch balance. Prentice et al. (2004) investigated walking from a level surface onto a ramp and found that even the smallest incline (3°) required adaptations to the swing limb trajectory (Prentice et al., 2004). We believe that the increased step time CoV found in our study could be the result of a similar proactive strategy to optimize the base of support during the rolling-hills terrain. Using the rolling-hills terrain condition, Sinitski et al. (2019) similarly found that healthy adults increased step time variability as well as step length variability compared to level walking (Sinitski et al., 2019). They also reported that participants increased step width during the rolling-hills condition compared to level walking. While they only investigated the rolling-hills as a single walking condition, we found increased step width to be specific to the uphill sections. However, the steps counted within the uphill and downhill sections can each be considered a transition step which reflect characteristics of both the current state as well as the upcoming state (Gottschall and Nichols, 2011). Therefore, it remains uncertain whether the increased step width is attributable to the current uphill section or in preparation for the upcoming downhill section. In either case, participants proactively modified their base of support to stabilize the COM when navigating the rolling-hills terrain.

The increased step width and double support time during uphill sections coincided with increased ML-MoS and decreased ML-MoS CoV. Vieira et al. (2017) similarly found increased ML-MoS during uphill sections, which increased stability, but their results showed decreased ML-MoS during downhill sections which we did not find (Vieira et al., 2017). Our results are somewhat different from Kawamura and Tokuhiro (1991) who found no step width increase during uphill sections (Kawamura and Tokuhiro, 1991). However, Kawamura's study examined a relatively narrow ramp which may have affected participants' ability to increase step width. The decrease we found in ML-MoS CoV may also be linked to uphill steps being consistently wider compared to level walking. In healthy individuals, decreased step width variability is thought to reflect greater active attention toward foot placement (Maki, 1997; Siragy and Nantel, 2018; Siragy et al., 2020). Additionally, increases in ML-MoS during perturbations may indicate a compensation response to mitigate destabilizing effects of the terrain, particularly as this finding was unique to the present study compared to previous investigations of ML-MoS during both uphill and downhill walking (Vieira et al., 2017). This demonstrates that the healthy young adults did adjust to the incline, even though the slope was minor, and successfully maintained stability.

### Mild Uphill and Downhill Slopes Required Spatiotemporal and Postural Modifications

Speed was slower for both uphill and downhill sections compared to level. This is somewhat similar to Kawamura and Tokuhiro (1991) which found a decrease in walking speed for both uphill and downhill conditions at 12°, but not at lower slopes (3, 6, 9°) (Kawamura and Tokuhiro, 1991). Our finding of decreased walking speed with slopes ranging from −3 to +3° may, therefore, be linked to the continuously varying nature of the rolling-hills terrain condition wherein a more cautious gait was employed for the duration of the terrain. Trunk posture was more backward during downhill sections and more forward during uphill sections, as hypothesized. Uphill walking is typically accompanied by a forward inclination of the trunk to aid in forward propulsion and stepping up (Leroux et al., 2002). Conversely, downhill walking is typically accompanied by a less forward trunk posture which assists in stepping down and the frictional demands on downhill slope (Leroux et al., 2002). The decreased walking speed and altered spatiotemporal and postural variables demonstrate that participants did make accommodations for the mild ( ≤ 3°) slopes encountered. Therefore, participants navigated the rolling-hills primarily by decreasing walking speed, but even the mild slopes caused spatiotemporal and postural changes.

### Active Arm Swing Required Proactive Strategies to Increase ML-MoS During Downhill Walking

We hypothesized that active arm swing may additionally perturb gait and require strategies that interact with those adopted for sloped walking. Instead, we found that the gait strategies used to manage active arm swing remained relatively consistent across slope conditions. However, the increase in ML-MoS seen with active arm swing compared to normal was only observed during downhill walking and corresponded to increased step width CoV. Hill and Nantel (2019) also found increased step width variability with active arm swing compared to normal during level walking (Hill and Nantel, 2019). They postulated that the more variable step width stemmed from the decreased coordination also found in the active arm swing condition and may have contributed to the concomitant increase in trunk local dynamic stability. The higher step width variability may demonstrate a proactive strategy to help stabilize the COM when walking with active arm swing, which was successful so far as to also increase ML-MoS in the downhill walking condition. This potentially shows that participants improved their mediolateral stability by varying their step width when managing the active arm swing.

### Arm Swing Effects Were Consistent Across Uphill and Downhill Sections of Rolling-Hills

We hypothesized that walking with arms held would lead to compound compensatory strategies during both uphill and downhill sections of the rolling-hills to increase stability. In both uphill and downhill sections, walking with arms held decreased speed compared to normal and active, which may indicate an extra level of caution when walking without arm swing. However, this did not appear to alter any strategies adopted during sloped walking. In fact, spatiotemporal differences from arm swing primarily existed with active arm swing compared to held and normal, with no significant differences between held and normal. For example, compared to held and normal, active arm swing increased step time, seemingly to preserve the coupling of arm-to-leg swing when the arms had further to swing (Bondi et al., 2017). This is further evidenced by the concomitant increase in step length during the active arm swing condition. It may be the case that the speed adjustment made by participants during the held condition was adequate to approximate normal walking stability and limit further need for spatiotemporal adjustments. Conversely, walking speed during active arm swing was not significantly different from normal but led to significant spatiotemporal differences from normal arm swing. Compared to normal arm swing, both held and active conditions caused distinct postural differences. Adopting a larger trunk angle with arms held projects the CoM further anteriorly, potentially reflecting an attempt to facilitate forward progression (Leroux et al., 2002). In contrast, the more upright posture (smaller trunk angle) during active arm swing may be an attempt to compensate for the forward-shifted CoM from increased anterior arm swing. While held and active arm swing illicited different strategies, these strategies remained separate from those used to navigate the slopes.

### Limitations

Both the “held” and “active” arm swing conditions could have led to increased attention compared to normal arm swing, which may approach the attentional requirements of some dual tasks. It is uncertain to what extent this affects the outcome parameters. The rolling-hills was a continuous slope condition wherein a range of angles were used rather than specific slope angles. While this is a more naturalistic terrain, it cannot provide insight to the strategies used to overcome specific surface angles or the extent of the spatiotemporal or postural strategies.

## Conclusion

Our study demonstrates that arm swing caused equivalent changes in all surface conditions. ML-MoS and step width CoV both increased within downhill sections of the rolling-hills terrain with the use of active arm swing compared to normal. This indicates that young, healthy participants may have improved their mediolateral stability by varying their step width when managing the active arm swing. Alternately, the increase in ML-MoS during uphill sections compared to level was accompanied by wider steps and longer double support time. Because stability increased during active arm swing with ongoing base of support adjustments and during sloped walking with consistently wider steps and longer double support, this demonstrates that different stepping strategies were used to manage active arm swing compared to a mild incline. Participants successfully navigated the rolling-hills by decreasing walking speed, but even the mild slopes caused spatiotemporal and postural changes. Specifically, the variability of the rolling-hills required participants to proactively modify their base of support to stabilize the COM. As this study tested healthy young adults, the current findings can be used as a baseline comparison in future investigations of other populations. Future research should focus on sloped walking in populations at risks of or with gait impairments (i.e., older adults or those with gait disorders).

## Data Availability Statement

The software and dataset produced and analyzed during this work are openly available in Zenodo at: https://doi.org/10.5281/zenodo.5608535.

## Ethics Statement

The studies involving human participants were reviewed and approved by the Institutional Review Board of the University of Ottawa and the Ottawa Hospital Research Ethics Board. The patients/participants provided their written informed consent to participate in this study.

## Author Contributions

JN: conceptualized and organized the research project. M-EM: data analysis—main analysis. TS and AH: data analysis—secondary analysis. JN, TS, and AH: data analysis—review and critique. M-EM, TS, and JN: statistical analysis—design. M-EM: statistical analysis—execution. JN, TS, and AH: statistical analysis—review and critique. M-EM: manuscript—writing of the first draft. JN, TS, and AH: manuscript—review and critique. All authors contributed to the article and approved the submitted version.

## Funding

This work was supported by the Natural Sciences and Engineering Research Council of Canada (NSERC) Discovery grant RGPIN-2016-04928, NSERC Accelerator supplement RGPAS 493045-2016 and by the Ontario Ministry of Research, Innovation and Science Early Researcher Award (ERA) 16-12-206.

## Conflict of Interest

The authors declare that the research was conducted in the absence of any commercial or financial relationships that could be construed as a potential conflict of interest.

## Publisher's Note

All claims expressed in this article are solely those of the authors and do not necessarily represent those of their affiliated organizations, or those of the publisher, the editors and the reviewers. Any product that may be evaluated in this article, or claim that may be made by its manufacturer, is not guaranteed or endorsed by the publisher.
