# Supplementary material for: Walking on Mild Slopes and Altering Arm Swing Each Induce Specific Strategies in Healthy Young Adults
Source: Front Sports Act Living. 2022 Jan 25;3:805147. doi: 10.3389/fspor.2021.805147 (PMC8821106; doi:10.3389/fspor.2021.805147)
Supplement: Supplementary file 1 [file Table_1.DOCX]

| **S1.** Average number of steps per participant in each arm/terrain condition. | | | | | | | | | | |
| --- | --- | --- | --- | --- | --- | --- | --- | --- | --- | --- |
|  | Uphill | | | Downhill | | | Level | | | |
| Participant | Held | Normal | Active | Held | Normal | Active | Held | Normal | Active |  |
| **1** | 6 | 3 | 4 | 8 | 9 | 6 | 14 | 15 | 11 |  |
| **2** | 5 | 7 | 7 | 9 | 8 | 7 | 16 | 12 | 13 |  |
| **3** | 6 | 7 | 6 | 7 | 8 | 7 | 17 | 15 | 13 |  |
| **4** | 6 | 8 | 6 | 8 | 6 | 5 | 17 | 18 | 19 |  |
| **5** | 6 | 6 | 5 | 9 | 8 | 7 | 15 | 13 | 12 |  |
| **6** | 7 | 6 | 5 | 6 | 9 | 5 | 16 | 11 | 18 |  |
| **7** | 7 | 7 | 5 | 9 | 8 | 7 | 14 | 16 | 10 |  |
| **8** | 7 | 7 | 4 | 6 | 7 | 4 | 17 | 13 | 12 |  |
| **9** | 8 | 6 | 6 | 9 | 7 | 8 | 17 | 21 | 17 |  |
| **10** | 10 | 8 | 6 | 10 | 8 | 5 | 17 | 17 | 14 |  |
| **11** | 7 | 6 | 6 | 6 | 6 | 6 | 14 | 15 | 12 |  |
| **12** | 6 | 6 | 7 | 4 | 7 | 7 | 22 | 14 | 15 |  |
| **13** | 7 | 5 | 6 | 8 | 7 | 6 | 14 | 14 | 11 |  |
| **14** | 8 | 6 | 6 | 5 | 6 | 7 | 13 | 11 | 11 |  |
| **15** | 7 | 4 | 6 | 7 | 7 | 6 | 15 | 10 | 10 |  |
| Mean | **6.87** | **6.13** | **5.67** | **7.4** | **7.4** | **6.2** | **15.9** | **14.3** | **13.2** |  |
| SD | **1.19** | **1.36** | **0.90** | **1.72** | **0.98** | **1.08** | **2.20** | **2.92** | **2.86** |  |

| **S2.** Average angle of sloped steps per participant in each arm condition (in degrees). | | | | | | |
| --- | --- | --- | --- | --- | --- | --- |
|  | Uphill | | | Downhill | | |
| Participant | Held | Normal | Active | Held | Normal | Active |
| **1** | 1.37 | 1.87 | 1.79 | -1.72 | -1.66 | -1.49 |
| **2** | 1.97 | 1.97 | 1.97 | -1.67 | -1.67 | -1.80 |
| **3** | 1.85 | 1.62 | 1.82 | -1.46 | -1.36 | -1.51 |
| **4** | 1.58 | 1.44 | 1.48 | -1.32 | -1.37 | -1.35 |
| **5** | 1.74 | 1.86 | 1.92 | -1.49 | -1.48 | -1.58 |
| **6** | 1.65 | 1.69 | 1.50 | -1.46 | -1.47 | -1.38 |
| **7** | 1.88 | 1.68 | 2.05 | -1.61 | -1.55 | -1.70 |
| **8** | 1.51 | 1.81 | 2.01 | -1.43 | -1.57 | -1.97 |
| **9** | 1.62 | 1.79 | 1.58 | -1.50 | -1.59 | -1.41 |
| **10** | 1.74 | 1.80 | 1.81 | -1.44 | -1.59 | -1.63 |
| **11** | 1.80 | 1.97 | 1.89 | -1.61 | -1.69 | -1.64 |
| **12** | 1.44 | 1.90 | 1.81 | -1.30 | -1.52 | -1.56 |
| **13** | 2.00 | 2.24 | 2.03 | -1.60 | -1.64 | -1.61 |
| **14** | 1.69 | 1.97 | 1.89 | -1.59 | -1.83 | -1.58 |
| **15** | 1.71 | 2.44 | 1.91 | -1.56 | -1.81 | -1.90 |
| Mean | **1.70** | **1.87** | **1.83** | **-1.52** | **-1.59** | **-1.61** |
| SD | **0.18** | **0.24** | **0.18** | **0.12** | **0.14** | **0.18** |

| **S3.** Correlations between ML-MoS, step width, and step width CoV for uphill and level walking in all three arm swing conditions (held, normal, active). | | | | | | | | | | | | | |
| --- | --- | --- | --- | --- | --- | --- | --- | --- | --- | --- | --- | --- | --- |
|  | Step width | | | | | | Step width CoV | | | | | | |
|  | Uphill | | | Level | | | Uphill | | | Level | | | |
|  | Held | Normal | Active | Held | Normal | Active | Held | Normal | Active | Held | Normal | Active |  |
| ML-MoS | .83** | .74** | .86** | .64* | .70** | .61* | -.41 | .27 | -.72** | .24 | -.46 | -.02 |  |
| Step width CoV | -.35 | .36 | -.65** | .12 | -.24 | -.01 | -- | -- | -- | -- | -- | -- |  |
| ** Correlation is significant at the .01 level (2-tailed).  * Correlation is significant at the .05 level (2-tailed). | | | | | | | | | | | | |  |

| **S4.** Correlations between step width CoV and ML-MoS during downhill walking between normal and active arm swing. | | |
| --- | --- | --- |
|  | ML-MoS | |
|  | Normal | Active |
| Step width CoV | -.21 | .39 |
